# Uremic Toxins and the Lung Alveolar Capillary Barrier: A Narrative Review

**DOI:** 10.3390/toxins18030126

**Published:** 2026-03-02

**Authors:** Saleh Kaysi, Maxime Taghavi, Alissa El Mourabi, Marie-Hélène Antoine, Eric De Prez, Joëlle Nortier

**Affiliations:** 1 Nephrology and Dialysis Department, Brugmann University Hospital, 1020 Brussels, Belgium; 2 Experimental Nephrology Laboratory, Faculty of Medicine, Université Libre de Bruxelles, 1070 Brussels, Belgium; 3 Nephrology and Dialysis Department, Grand Hôpital de l’Est Francilien, 77100 Meaux, France; 4 Faculty of Medicine, Université Catholique de Louvain, 1348 Brussels, Belgium

**Keywords:** uremic toxins, lung, inflammation, oxidative stress, endothelial cells, epithelial cells, alveolar capillary barrier, lung congestion, lung edema

## Abstract

Introduction: Uremic toxins have been shown to cause adverse pulmonary effects by inducing endothelial and epithelial dysfunction, disrupting the alveolar-capillary barrier, and increasing inflammation and oxidative stress. This article reviews these effects with a specific focus on chronic kidney disease and the mechanisms by which uremic toxins affect lung tissue. Methods: A narrative review was conducted using keywords related to uremic toxins and lung injury to search the PubMed database. An advanced literature review was conducted in PubMed to identify studies explaining the mechanisms underlying lung pathophysiology in chronic kidney disease (CKD), with particular focus on CKD-induced pulmonary epithelial and endothelial dysfunction. Additionally, to highlight the pathological processes of lung congestion in CKD, studies on CKD-induced dysfunction of the alveolar-capillary barrier were retrieved. Studies published up to November 2025 were evaluated. Results: A total of 148 articles were reviewed in full text. Uremic toxins negatively impact lung tissue structure and function through multiple mechanisms, including oxidative stress, inflammation, and direct effects. Uremic toxins appear to share signaling pathways in endothelial cells, including those linked to Mitogen-activated protein kinases (MAPK), the Aryl Hydrocarbon Receptor (AhR), the receptor for advanced glycation end products (RAGE), and pro-inflammatory transcription factors such as nuclear factor κB (NF-κB). Additionally, oxidative stress acts as a pro-inflammatory signal shared by several uremic toxins. The mechanisms behind the harmful interactions between CKD and lung disease are mostly unknown, although more evidence exists for acute kidney injury (AKI). Conclusions: Chronic kidney disease, which leads to the buildup of uremic toxins, negatively affects the lungs. Overall, the accumulation of uremic toxins in CKD impairs endothelial and epithelial cells and the alveolar capillary barrier. Further research is needed to understand the specific mechanisms underlying these effects and to identify therapeutic options to protect the lungs in these patients.

## 1. Introduction

More than 140 uremic toxins (UTs) have been identified as elevated in cases of kidney dysfunction [1,2,3]. Over 100 of them have been described and classified by the European Uremic Toxin (EUTOX) Working Group [2]. These toxins are linked to higher cardiovascular event risk and mortality in chronic kidney disease (CKD) patients.

Historically, pulmonary complications in CKD were primarily attributed to volume overload and uremia, manifesting as pulmonary edema and pleuritis. With advances in understanding, it is now clear that CKD is associated with a spectrum of lung disorders, including restrictive lung disease, pulmonary hypertension, and increased susceptibility to infections [4,5].

While both acute kidney injury (AKI) and CKD impact the lungs, most research has concentrated on the connection between AKI and acute lung injury (ALI). The pulmonary effects of uremia have been less extensively studied, although CKD patients demonstrate a significantly higher prevalence of respiratory illnesses [4,5]. Studies indicate that renal disease increases susceptibility to lung dysfunction, with declining kidney function correlating with increased obstructive and restrictive abnormalities [6]. Restrictive lung disease prevalence is particularly elevated in stage 5 CKD with a glomerular filtration rate (GFR) less than 15 mL/min and represents the most common pulmonary dysfunction in end-stage kidney disease (ESKD) requiring maintenance hemodialysis (HD) [7]. Furthermore, those with CKD have lower pulmonary function, respiratory, and peripheral muscle strength than the general population, negatively impacting quality of life [8]. In a community-based cohort followed over 14 years, those with renal disease experienced a faster decline in lung function compared with those who had normal kidney function [9]. In rats, six months after unilateral ureteral obstruction (a CKD model), lung tissue showed fibrosis with increased collagen deposition [10].

Volume overload can negatively impact lung function by worsening interstitial edema, impairing gas exchange and oxygenation. However, this alone does not fully explain respiratory problems in kidney disease [11,12]. In AKI, cardiogenic pulmonary edema, caused by elevated capillary hydrostatic pressure, can be relieved by fluid removal via ultrafiltration or diuresis, whereas non-cardiogenic edema, resulting from inflammation-related hyperpermeability, responds only partially to fluid removal [13]. Aggressive fluid removal through HD has been linked to increased mortality among those with kidney failure [14].

Pulmonary hypertension is increasingly recognized as a common and prognostically important complication of chronic kidney disease, driven by mechanisms such as volume overload, left ventricular dysfunction, pulmonary vascular remodeling, and endothelial injury [9].

In HD patients, p-Cresyl Sulfate (pCS) is an independent risk factor for carotid atherosclerotic plaque [15]. Advanced glycation end products (AGEs) are protein-bound uremic toxins significantly elevated in CKD. AGEs are proteins or protein breakdown products that undergo post-translational modification upon exposure to sugars [16]. The transmembrane receptor for AGEs (RAGE) is a cell surface marker involved in cell migration, adhesion, and oxidative stress [17].

UTs can lead to endothelial dysfunction, inflammation, and oxidative stress, thereby increasing cardiovascular risk. It is reasonable to assume that HD will clear UTs and reduce their levels, depending on their dialyzability. However, pre-dialysis levels of UTs rely only partially on their dialyzability. The levels of water-soluble trimethylamine N-oxide (TMAO), as well as Hippuric acid, Indoxyl Sulfate (IS), and Indole-3-Acetic acid (IAA), which are protein-bound, are all significantly higher in HD patients compared to CKD stage 5 patients [18]. Additionally, HD patients exhibit higher levels of Asymmetric Dimethylarginine (ADMA) and lower levels of Symmetric Dimethylarginine (SDMA) compared to CKD stage 5 patients [18]. Furthermore, plasma pCS levels do not differ between patients with CKD stages 4–5 and those undergoing HD [19]. This demonstrates that various factors, including diet, residual kidney function, the type of renal replacement therapy, and inflammation, significantly influence pre-dialysis levels of UTs [20]. Table 1 presents selected UTs and their characteristics.

Numerous mechanisms may contribute to uremic lung injury (ULI), including neutrophil activation, vascular hyperpermeability, dysregulation of salt-water transporters, and cytokine overexpression [21,22,23,24]. However, most experimental models involve animals with AKI rather than CKD. Our current understanding of the mechanisms underlying pulmonary complications in CKD is limited. Despite documented prior associations, the adverse effects of uremic toxins on the lungs remain unclear.

In this review, we primarily examine the impact of UTs and CKD on the lungs, although we also include evidence on AKI-associated lung injury. Our focus will be on how CKD causes lung injury. We will provide an overview of the effects of UTs and CKD on inflammation, oxidative stress, alveolar epithelial cells, pulmonary vascular endothelial cells, and the alveolar-capillary barrier.

## 2. Materials and Methods

This is a narrative review.

### 2.1. Search Strategy

An advanced literature review was conducted in PubMed to identify studies explaining the mechanisms underlying lung pathophysiology in CKD, with particular focus on CKD-induced pulmonary epithelial and endothelial dysfunction. Additionally, to highlight the pathological processes of lung congestion in CKD, studies on CKD-induced dysfunction of the alveolar-capillary barrier were retrieved. Studies published up to November 2025 were evaluated. The Supplementary Materials (Table S1) provides an overview of the terms and conditions used in the literature search.

PubMed was the sole database used to conduct this narrative review, as it provides broad coverage of the biomedical literature and was considered sufficient to support a comprehensive assessment of the topic.

### 2.2. Study Selection Criteria

Duplicates, review papers, poster abstracts, editorials, letters, books, and papers not written in English were excluded, along with studies focusing on CKD but lacking mechanistic information. Studies older than 2000 and those not addressing the specific impact of uremic toxins on pulmonary structure were excluded. Only PubMed was used to screen for studies, excluding gray literature publications, since the review included only validated data.

The authors used keywords and search terms (MeSH) to search PubMed in a consistent, standardized way for relevant articles. These MeSH terms are provided in Supplementary Materials (Table S1). Two of the authors, S.K. and A.M., independently reviewed the search results and applied the inclusion and exclusion criteria. Each then evaluated the titles and abstracts of the remaining articles to decide which to examine in full text and include in the review. The lists from the two authors were then cross-matched. Articles selected by both authors were automatically included. Unmatched articles were then reviewed by a third author, M.T., who determined whether to include each article.

### 2.3. Data Extraction

To summarize the pathophysiological effects of uremia or UTs on the lungs, studies were organized by toxin and its primary lung effects. The key effects identified include inflammation, oxidative stress, injury to endothelial or epithelial cells, abnormal alveolar fluid clearance, and increased permeability of the alveolar-capillary barrier. To understand the molecular mechanisms underlying uremic toxin-induced lung dysfunction, signaling pathway information was collected from both in vivo and in vitro studies. Furthermore, data on lung function markers or information on uremic toxin-related signaling pathways were derived from patient studies.

While it is beyond the scope of this review to weigh the evidence from studies based on their experimental model (human, animal, or in vitro), a table is included to demonstrate these models (Table 2).

## 3. Results

Using predefined literature search terms (Table S1), our search initially included 1289 articles identified via PubMed. After removing non-English publications and duplicates, and conducting abstract screening, 1140 publications were excluded as out of scope.

**Figure 1:**
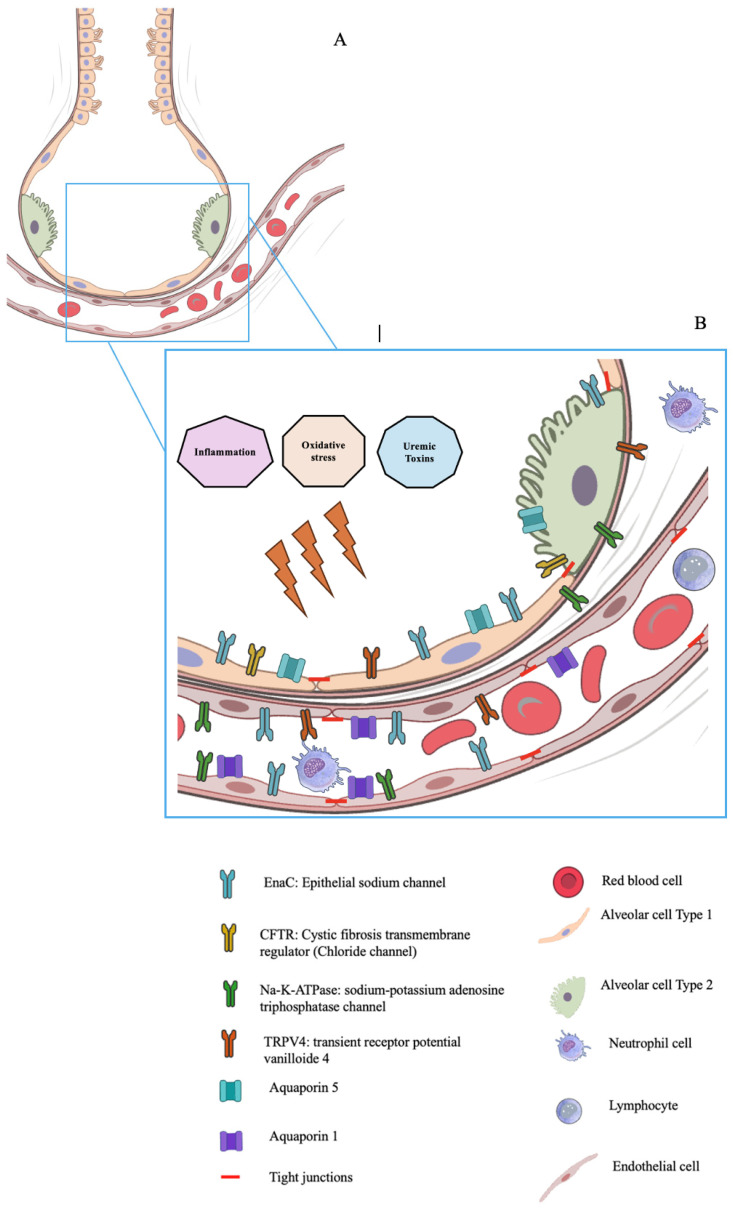
(**A**) The structure of the pulmonary alveoli. (**B**) The different structures of the alveolar-capillary barrier, including the main channels and proteins that may be affected by uremic toxins. The impact of uremic toxins on these structures can be direct or mediated through inflammation and oxidative stress. Uremic toxins can impact endothelial and epithelial cells, leading to alterations in their structure, surface expression of channels and aquaporins, or inducing apoptosis.

Figure 1 shows the main alveolar capillary barrier structures, which may be affected by UTs. The full texts of the remaining 148 papers were examined. In total, 148 publications were included in the review.

### 3.1. UTs and Inflammation

Chronic kidney disease is considered a persistent, low-grade, systemic inflammatory condition with disrupted cytokine homeostasis [68]. Research on the effects of cytokine release and reduced excretion, particularly regarding lung disease, remains limited. A cross-sectional study examining lung dysfunction and its link to inflammation across different GFR categories found that inflammation plays a central role in pulmonary-renal interactions [7].

The mechanisms through which UTs promote inflammation are interconnected and complex. In CKD patients, systemic inflammation is indicated by elevated levels of inflammatory markers, such as C-reactive protein (CRP) and fibrinogen, as well as pro-inflammatory cytokines such as Interleukin-1 (IL-1), Interleukin-6 (IL-6), and Tumor Necrosis Factor-alpha (TNFα). These markers correlate with decreased kidney function [40]. Members of the Tumor Necrosis Factor (TNF) family, such as TNF-α from one side, and its soluble receptors (sTNF-R1 and sTNF-R2) from the other, are key factors in the inflammatory response to UTs [69].

The accelerated atherosclerotic process in CKD may involve several interconnected mechanisms, including oxidative stress, endothelial dysfunction, and vascular calcification, within a constant environment of low-grade inflammation characterized by impaired neutrophil and T-cell function and a dysregulated cytokine network [68].

Those on maintenance HD may experience chronic systemic microinflammation [29], driven by multiple factors, including the accumulation of proinflammatory interleukins and chemokines [70]. CKD patients exhibit systemic, chronic, low-grade inflammation and increased oxidative stress, even in early stages, as evidenced by elevated circulating inflammatory proteins (e.g., CRP, IL-6) and oxidative stress biomarkers [52]. Microinflammatory status, endothelial damage, and elevated Vascular Endothelial Growth Factor (VEGF) levels were observed in those not on HD and in those undergoing peritoneal dialysis (PD). However, these changes were most pronounced in ESKD on chronic HD compared to healthy controls [50].

Long-term micro-inflammation can lead to changes in cardiovascular structures and functions, eventually resulting in cardiovascular disease [71,72]. Uremia promotes vascular calcification through a signaling pathway involving TNF-α, IL-6, and the cytokine signaling axis [67]. Inflammation measured by CRP or IL-6 is a significant predictor of major cardiovascular events and may explain the remaining cardiovascular risk even with statin therapy [73].

It is well established that inflammation and oxidative stress contribute to endothelial dysfunction [74]. High levels of Reactive Oxygen Species (ROS) are associated with increased expression of Monocyte Chemoattractant Protein-1 (MCP-1) and Intercellular Adhesion Molecule-1 (ICAM-1). These are key mediators of inflammatory cell recruitment and adhesion, activated by ROS-dependent signaling through the Nuclear Factor κB (NF-κB) pathway [49,63,75].

IAA induces endothelial inflammation and oxidative stress through Aryl Hydrocarbon Receptor (AhR)-dependent signaling mechanisms [35].

In AKI models, systemic inflammatory responses triggered by factors such as TNF-α, Interleukin-1β (IL-1β), IL-6, and macrophage inflammatory protein (MIP-1), which are released into the circulation during renal ischemia–reperfusion (RIR), can directly damage the lungs [66]. Several soluble inflammatory mediators associated with AKI induces remote lung inflammation, such as IL-1β, Interferon Gamma (IFN-γ), IL-6, TNF, and Transforming Growth Factor Beta (TGF-β), are also elevated in the serum, kidney, and lung of patients with CKD [76].These inflammatory mediators play essential roles in the pathophysiology of both acute and chronic lung diseases [77].

Hyperphosphatemia leads to increased ROS production, which, in turn, promotes the production of inflammatory proteins, including TNF-α, ICAM-1, IL-6, IL-1ß, and IL-8, thereby contributing to increased inflammation [48].

In HD, elevated levels of Fibroblast Growth Factor (FGF-23) are associated with inflammation [78]. Osteoprotegerin, a soluble receptor in the TNF superfamily that inhibits osteoclast differentiation and activation by blocking the interaction between Receptor Activator of Nuclear Factor (RANK) and Receptor Activator of Nuclear Factor Ligand (RANKL), has been suggested as an early marker of coronary calcification among pro-calcifying stimuli. Elevated osteoprotegerin levels correlate with increased risk of all-cause and cardiovascular mortality. Recent research also links osteoprotegerin to the development of vascular inflammation and endothelial dysfunction caused by angiotensin II [79].

Furthermore, UTs include several inflammatory cytokines (IL-6, TNFα, IL-1β, IL-18) and a chemokine (IL-8), which are considered secondary uremic toxins with a very low dialyzability [80].

HD might be expected to reduce inflammation by removing uremic toxins. However, some inflammatory triggers are directly linked to HD treatment [81]. In HD patients, IL-18 plasma levels are twice as high before dialysis compared to control subjects and increase further by the end of the dialysis session [82]. Notably, plasma IL-8 levels are elevated even before the HD stage [83].

A recent study demonstrated that hypoxemia in AKI is partly caused by impaired pulmonary blood flow due to retained and nondeformable intravascular neutrophils, revealing a new mechanism of uremia-mediated lung injury [43].

### 3.2. UTs and Oxidative Stress

Uremia contributes to oxidative stress [84], and as CKD progresses, oxidative stress levels increase [85]. Pulmonary oxidative stress is associated with kidney injury, suggesting a mechanistic link between the kidney and lung in disease states [51].

Mice fed an adenine diet for one month to induce CKD showed increased lung injury and fibrosis on histology, along with significant neutrophil infiltration in the alveolar and bronchial walls, accompanied by evidence of heightened oxidative stress [51]. Additionally, Nuclear Erythroid-Related Factor 2 (Nrf2), a transcription factor that induces antioxidant enzyme expression during oxidative stress, was increased in the lungs of these mice, suggesting that oxidative stress occurs in the lungs of mice with CKD [45].

CKD causes heightened oxidative stress by producing excessive ROS and Reactive Nitrogen Species (RNS), along with depletion of antioxidant responses [86]. Oxidative stress reduces nitric oxide (NO) bioavailability, promotes lipid oxidation, and contributes to inflammation [87].

Protein-bound UTs, such as pCS and IS, negatively affect endothelial cells by increasing oxidative stress and ROS formation [34,38].

IS increases oxidative stress through various pathways, including activating the Nicotinamide Adenine Dinucleotide Phosphate (NADPH) oxidase [34,49], reducing levels of the antioxidant glutathione [34], and triggering the AhR pathway [41]. It also decreases NO production and endothelial Nitric Oxide Synthase (eNOS) phosphorylation in endothelial cells [63]. IAA increases the production of endothelial ROS [35]. pCS increases ROS in endothelial and smooth muscle cells in vivo and in vitro [15]. Non-specific intracellular ROS contributed to pCS-induced alveolar cell death [30]. pCS-induced intracellular inflammatory signaling pathways are diverse and intermixed.

Hyperphosphatemia is associated with inflammation and oxidative stress, which contribute to endothelial dysfunction [25,48,53,88]. Phosphate levels that deviated above or below the physiological value of 1 mmol/L resulted in decreased NO production in endothelial cells [53].

Markers of oxidative stress are inversely associated with endothelium-dependent vasodilation in patients with CKD, independent of traditional atherosclerosis risk factors such as gender, age, blood pressure, and diabetes [27].

It is worth noting that chronic uremia has a distinct impact compared to AKI. Unlike chronic uremia, AKI does not alter superoxide production or neutrophil apoptosis [65].

### 3.3. UT and Endothelial Cells

Under physiological conditions, the vascular endothelium regulates processes such as vascular tone, vascular permeability, leukocyte recruitment (and thus inflammation), platelet adhesion and aggregation, activation of the coagulation cascade, and fibrinolysis [89].

Endothelial dysfunction is a prominent characteristic of cardiovascular disease in renal disease, leading to higher morbidity and mortality [90]. It results from severe dysregulation of uremic and inflammatory mediators [50]. and underlies both atherosclerosis and vascular stiffness, thereby increasing cardiovascular disease risk [91]. The development and progression of atherosclerotic lesions and vascular stiffness are accelerated in this population [92].

Additionally, atherosclerosis and intimal hyperplasia frequently occur in patients with uremia and are closely linked to vascular endothelial dysfunction [93].

pCS and IS reduce endothelial proliferation and wound healing [33]. Oxidative stress, inflammation, cell death, and reduced proliferation are central to IS-induced endothelial dysfunction [34,38,41,42,44,49,63,75]. Increased IS levels induce endothelial cell apoptosis [44].

Protein-bound uremic toxins, such as pCS and IS, negatively affect endothelial cells. They enter cells via Organic Anion Transporters (OATs), primarily OAT1 and OAT3, thereby activating various signaling pathways that ultimately lead to endothelial dysfunction in CKD [37,94].

Vascular Endothelial Growth Factor (VEGF) is a growth and survival factor for endothelial cells [95]. Autocrine VEGF production is essential for maintaining the proper function and survival of endothelial cells [96].

Endothelial cells produce VEGF in response to stressors, including inflammation and hypertension. Its levels increase in hypertension, and treatment normalizes them [97], making VEGF a good marker of endothelial dysfunction. Prolonged endothelial stress caused by UTs can lead to maladaptive endothelial responses, excessive VEGF production, and depletion of regenerative ability, ultimately resulting in vascular disease and cardiovascular complications [98]. Renal disease is characterized by disrupted VEGF production and maladaptive angiogenesis, linked to inflammation and increased morbidity and mortality in HD [64]. Furthermore, VEGF is significantly upregulated in response to uremic mediators in HD serum [29].

TNF signaling is a key component within the altered cytokine network of uremia-induced endothelial cell dysfunction [70]. Endothelial VEGF expression and angiogenesis depend heavily on both TNF-α and sTNF-R1 concentrations [29]. TNF signaling plays a key role in regulating endothelial VEGF production and angiogenic maladaptation, and the TNF-α/sTNF-R1 ratio in uremic patients’ serum indicates endothelial dysfunction [69,99]. In addition, in these patients, proliferation of vascular endothelial cells is inhibited, and inflammation is increased [45].

In CKD patients, Cyanate levels can rise to three times the normal levels. Cyanate significantly contributes to endothelial dysfunction and vascular inflammation [36,100].

Connexins are essential for maintaining endothelial monolayer integrity and stabilizing contacts among endothelial cells. During the early stages of increased vascular endothelial cell permeability, connexins are damaged, a crucial factor in endothelial dysfunction. Additionally, the downregulation of Zonula Occludens-1 (ZO-1) and Vascular Endothelial Cadherin (VE-cadherin) expression serves as markers of increased endothelial permeability and vascular dysfunction [101]. ZO-1 expression in the arteries of uremic patients was lower than that in the arteries of healthy volunteers [45].

Receptors involved in UT effects include RAGE, Nuclear Factor kappa-B (NF-κB) signaling, and Glutathione S-transferase-1 (GSTM-1), which is a downstream gene in the AhR signaling pathway. These were responsible for abnormal endothelial expression of adhesion molecules [102].

The AGE-RAGE signaling pathway contributes to uremic toxin-induced endothelial dysfunction. AGEs inhibit eNOS expression [17]. Additionally, AGEs may induce endothelial inflammation and oxidative stress independently of RAGE signaling and impair Endothelial Progenitor Cell (EPC) adhesion and proliferation, further contributing to endothelial dysfunction [17,103].

pCS is associated with cardiovascular disease in hemodialysis [104]. Research has demonstrated a connection between pCS, oxidative stress, and endothelial dysfunction. In addition to AGEs, pCS also binds to RAGE [54]. pCS stimulation of endothelial cells induced ROS formation via Nicotinamide Adenine Dinucleotide Phosphate Oxidase (NADPH oxidase), independent of RAGE, and was primarily driven by free pCS rather than protein-bound forms [60]. In hemodialysis, increases in free and total pCS correlate with the number of Endothelial Microparticles (EMPs) and shed EMPs [15]. Additionally, pCS induces ROS-mediated apoptosis of endothelial cells [38].

Elevated serum phosphate levels contribute not only to vascular calcification but also to inflammation and endothelial dysfunction [55].

High phosphate levels, common in CKD-related mineral bone disorders, may lead to endothelial dysfunction by altering endothelial cell morphology, reducing cell viability, and promoting senescence [105]. Hyperphosphatemia and hypophosphatemia negatively affect endothelial cell viability [53].

The phosphaturic hormone FGF23 is also significantly elevated in CKD and impairs endothelium-dependent vasorelaxation. Underlying mechanisms include increased ROS production and decreased NO bioavailability, most likely independent of the FGF23 cofactor Klotho [56].

Klotho has been shown to decrease endothelial permeability and apoptosis [106].

Klotho expression is downregulated in CKD due to systemic inflammation, primarily via inflammatory cytokines that activate NF-κB [107]. Klotho reduces endothelial inflammation by inhibiting NF-κB nuclear translocation, decreasing oxidative stress, increasing endothelial NO production, and protecting endothelial cells from uremia-induced senescence [108].

Circulating EPCs derived from bone marrow aid in vascular repair. Their levels in peripheral blood are linked to endothelial function and cardiovascular risk. Those with renal disease exhibit markedly lower EPC counts compared to healthy individuals [32].

### 3.4. UTs and Epithelial Cells

Physiologically, pulmonary edema can result from increased hydrostatic pressure in the pulmonary vasculature or elevated vascular permeability. The resolution of alveolar edema depends on the active removal of sodium and water from the distal air spaces across the lung epithelial barrier [109].

Sodium crosses the apical membranes of alveolar epithelial cells via both amiloride-sensitive and non-sensitive cation channels, including the amiloride-sensitive Epithelial Sodium Channel (ENaC). It is then moved into the interstitial space through the Sodium-Potassium ATPase (Na+/K+/ATPase) on the basolateral membrane pump [110].

Active alveolar epithelial transport plays a vital role in maintaining lung fluid balance. Type I cells exhibit the highest known water permeability of any mammalian cell type, which likely accounts for the lung’s high water permeability [111].

Chronic regulation of membrane channels and pumps is mediated not only by changes in their synthesis but also by variations in their rates of degradation [112].

The alveolar epithelium is notably resistant to injury, especially when compared to the nearby lung tissue endothelium [113]. Injury to the alveolar epithelium can compromise the integrity of the alveolar barrier or decrease ion transport pathways, thereby reducing net alveolar fluid reabsorption and increasing alveolar edema.

Reactive oxygen or nitrogen species can decrease alveolar fluid transport. These species, at concentrations similar to those released by activated macrophages, reduce the activity of alveolar cell sodium channels [39].

Specialized water-transporting proteins, Aquaporins (AQPs), have been localized in the lung: AQP1 in microvascular endothelial cells and AQP5 at the apical membrane of alveolar epithelial cells [58]. Deletion of AQP1 or AQP5 caused a tenfold decrease in osmotically driven water transport between the airspace and capillary compartments [46].

The impact of UT on AQPs remains incompletely established. Decreased expression of lung AQP5 and AQP1 has been observed in AKI [23,47,62].

pCS exhibits pro-oxidant properties in epithelial cells by increasing NADPH oxidase activity. This mechanism is similar to that responsible for the renal toxicity of IS [114]. IFN-γ increases permeability in lung epithelial cells [28].

In human lung epithelial cells, IL-13 increased epithelial permeability and decreased ZO-1 and Occludin expression [26]. Increased IL-10 expression reduced the rise in lung epithelial permeability in a lung injury model [115].

### 3.5. UT and the Alveolar-Capillary Barrier

Two distinct barriers make up the alveolar/capillary barrier: the microvascular endothelium and the alveolar epithelium, as shown in Figure 1.

Even when lung endothelial injury occurs, the alveolar epithelial barrier can remain normally impermeable to proteins and maintain normal or increased fluid transport capacity, thereby confining pulmonary edema to the interstitium [116,117].

The lung epithelium serves as the primary barrier separating the air spaces from the fluid-filled vascular and interstitial compartments, thereby providing high resistance to the movement of fluids and solutes. This pulmonary epithelial barrier not only restricts the passive movement of aqueous fluids and solutes but also facilitates the removal of airspace fluid via active solute transport across the epithelium from the alveolar space [118].

Lung tissues from CKD mice exhibit reduced alveolar cell numbers, diffusely dilated alveolar spaces, plasma leakage, interstitial edema, and increased leukocyte recruitment, supporting the evidence of alveolar–capillary injury and suggesting that UTs impair barrier integrity [30].

Generally, the lung epithelial barrier is much less permeable than the endothelial barrier. Endothelial cells exposed to uremia sera demonstrate significantly higher permeability than healthy cells, with UTs increasing endothelial cell permeability in a time-dependent manner [45].

Serum from patients with uremia increased permeability in pulmonary microvascular endothelial cells [61]. Endothelial barrier permeability is mainly maintained by a balance between adhesive forces at endothelial cell–cell junctions and contractile forces generated within the endothelial cytoskeleton [119].

In an animal model of renal ischemia–reperfusion, the pulmonary structure was almost completely damaged, with notable cell infiltration and significant erythrocyte leakage into the alveolar space [31]. In vitro, the permeability coefficient (Pc) of the pulmonary microvascular endothelial cell (PMVEC) monolayer treated with RIR serum increased markedly [31].

Resolution of pulmonary edema occurs through active sodium transport across the alveolar epithelium via apical and basolateral sodium channels, a process known as alveolar fluid clearance [120].

The transport of sodium across the alveolar epithelium through apical sodium channels, such as ENaC, creates an osmotic gradient that causes water to move passively from the air spaces into the interstitium. This process (alveolar fluid clearance) is essential for maintaining effective gas exchange in the normal lungs. ALI patients with intact alveolar fluid clearance experienced lower morbidity and mortality compared to those with impaired clearance [59].

The ENaC and Na-K-ATPase are sodium cotransporters that play key roles in this process [109]. Several studies suggest that dysregulation of lung salt and water channels after AKI plays a key role in ALI [121]. AQP5 is responsible for the majority of water transport across the apical membrane of alveolar epithelial cells [46]. Few studies suggest that renal failure downregulates AQP5, the pulmonary epithelial sodium channel, and Na+/K+-ATPase, which may contribute to lung function abnormalities and increased susceptibility to lung injury [23]. In rat models of either RIR or bilateral nephrectomy, researchers observed a downregulation of pulmonary ENaC, Na-K-ATPase, and AQP5 [23]. Others demonstrated increased pulmonary vascular permeability in rats after RIR, along with enhanced expression of adhesion molecules and neutrophil recruitment into the lung [22]. AKI dysregulates pulmonary AQP5 expression, and IS may mediate renopulmonary crosstalk [62].

No studies have directly examined the impact of UT on the tight junction function and structure in pulmonary endothelial or alveolar epithelial cells, so we include evidence from research on these structures in the brain and intestinal tissues, assuming similarity to lung tissue.

In the brain, both chronic and acute renal injury disrupt the blood–brain barrier (BBB) [122,123,124]. Brain cortex tissues from CKD rats showed significant reductions in ZO-1, junctional adhesion molecules (JAMs), and Occludin levels [125].

Expressions of the essential tight-junction protein Claudin-5 and the adherens-junction protein Platelet Endothelial Cell Adhesion Molecule-1 (PECAM-1) were reduced in the brain endothelial cells of CKD mice. Urea dose-dependently decreased both Claudin-5 and PECAM-1 expression in the mouse brain endothelial cell [126].

Renal disease leads to obvious intestinal barrier dysfunction [127]. Some studies have initially explored the hypothesis that certain UTs, such as IS, can damage intestinal TJ proteins and intestinal barrier function [128,129].

The intestinal barrier comprises epithelial cells and the apical junctional complex [129]. The apical junctional complex consists of the TJ which includes three elements: I- adhesive transmembrane proteins such as Occludin and Claudin family of proteins which link the plasma membranes of adjacent cells to form a barrier to the diffusion of fluids and solutes; II- the cytosolic proteins, i.e., the Zonula Occludens (ZO) protein family, which serve as anchors by binding simultaneously to the intracellular domains of Occludin and Claudins and to the peri-junctional actin-myosin ring; and III- the peri-junctional ring of actin and myosin [130].

Few studies have demonstrated significant depletion of colonic epithelial TJ proteins and their role in the development of endotoxemia and systemic inflammation [57,131]. Urea significantly increases intestinal permeability and reduces TJ protein expression, including ZO-1, Occludin, and Claudin-1 [127].

Pre-dialysis plasma from ESKD increased intestinal epithelial cell permeability. This effect was significantly less pronounced in cells exposed to post-dialysis plasma than in those exposed to pre-dialysis plasma [131]. There was a significant loss of colonic epithelial TJ in rats with renal disease caused by either 5/6 nephrectomy or chronic tubulointerstitial nephritis [57].

When comparing data from epithelial monolayers exposed to media with post-dialysis plasma to those incubated with pre-dialysis plasma, there was a significantly less severe reduction in ZO-1, Claudin-1, and Occludin proteins [132].

Uremia-induced disruption of intestinal TJs and barrier function is partly mediated by urea [131]. Pro-inflammatory cytokines produced during the inflammatory response, including TNF-α, IFN-γ, IL-1β, and IL-12, disrupt the intestinal TJs, thereby increasing TJs permeability [57].

This phenomenon may explain the presence of endotoxemia and increased intestinal permeability to high-molecular-weight polyethylene glycol in patients with advanced CKD, indicating a direct link between the severity of systemic inflammation and plasma endotoxin levels in the ESKD population [133].

Using polyethylene glycol molecules of increasing molecular weight, it was demonstrated that the TJ barrier defect induced by IFN-γ in intestinal epithelial cells was accompanied by a greater increase in permeability to larger molecules [134].

Together, these findings demonstrate the disruptive effect of uremia on both the intestinal epithelial barrier and the blood–brain barrier. This may support that similar effect is induced in the endothelial and epithelial pulmonary cells. Table 3 summarizes main molecular changes in the alveoli in uremic conditions.

## 4. Discussion

UTs, inflammation, and endothelial dysfunction are recognized as key contributors to the development and severity of cardiovascular disease in renal disease [135].

Since CKD affects the lungs, we reviewed uremic toxin-induced pulmonary dysfunction and its underlying mechanisms through a narrative review.

UTs share signaling pathways in endothelial cells, including those linked to MAPK, AhR, the RAGE receptor, and pro-inflammatory transcription factors like NF-κB. Additionally, ROS acts as pro-inflammatory signals shared by multiple UTs. Various uremic toxins, such as IS, phosphate, Cyanate, AGEs, and Uric acid, decrease eNOS expression and/or activity, reducing NO production [17,36,53,63]. CKD is characterized by chronic inflammation and oxidative stress, which damage the endothelium, impair endothelial cell function, reduce NO production, and impair vasodilation [136].

The mechanisms underlying the harmful interactions between CKD and lung disease are largely unknown, and most current knowledge is correlative, with limited mechanistic evidence from animal models [137]. More evidence is available for AKI, as it is a disease process that disrupts alveolar-capillary barrier integrity and can overwhelm the lung’s ability to regulate fluid balance [138].

Since there is currently no universal method that effectively removes all types of (protein-bound) UTs, further research is necessary to enhance UT removal and improve lung health in CKD patients.

Both PD and HD patients exhibit increased systemic inflammation and oxidative stress, which can contribute to pulmonary and alveolar dysfunction. HD is associated with acute changes in pulmonary function, including transient hypoxemia and impaired alveolar ventilation during dialysis sessions, likely due to leukocyte sequestration and microvascular injury in the lung, as well as hypoventilation and ventilation-perfusion mismatch [139]. HD patients have higher exhaled nitric oxide (FeNO) levels, indicating greater oxidative stress and inflammation than PD patients [140].

Lung recovery after kidney transplantation is expected due to the resolution of uremia and restoration of kidney function, which reduces cytokine production and facilitates their clearance, potentially leading to decreased lung inflammation and improved alveolar integrity. However, no study has examined the direct impact of kidney transplantation on the restoration of the alveolar capillary barrier’s structure and function. In addition, immunosuppressants and other medications used to maintain kidney graft function may affect the lungs and could counteract the positive effects of kidney function restoration. Furthermore, the graft function level is not always optimal, and many transplanted patients maintain some degree of kidney dysfunction.

Although the impact of CKD on the lungs has been clinically demonstrated, there is limited evidence describing the underlying pathophysiological mechanisms behind this effect. The few explanations available are mainly based on AKI-induced lung injury, suggesting both pharmacodynamic and pharmacokinetic etiologies. This highlights the knowledge gap in understanding how CKD affects alveolar capillary structure and function. While AKI and CKD share the accumulation of UT as a common feature, it is obvious that AKI is a different disease state than CKD, making it difficult to suppose that they have the same pathophysiological mechanism on the lung. In the acute condition, the increase in UT levels is sharp, accompanied by other factors perturbation according to the cause of AKI, while it is gradual and usually isolated in chronic conditions. Furthermore, the interstitial space compliance is different in acutes situation from the chronic ones, resulting in different impacts of the same amount of volume overload.

We think that CKD, in addition to being a chronic inflammatory state, reduces cytokine clearance, leading to their accumulation in the patient’s body. This buildup of inflammatory mediators, along with the direct effects of UT, causes oxidative stress in lung tissue, which damages the structure and function of the alveolar-capillary barrier.

## 5. Future Directions and Therapeutic Implications

Lung complications due to CKD have an impact on these patients’ quality of life, morbidity, and mortality. To effectively prevent and manage these complications, a thorough understanding of how CKD produces these effects is essential.

Better, more practical, and objective methods to evaluate volume status, pulmonary arterial pressure, and lung congestion are needed to manage volume overload and reduce the hydrostatic pressure that drives fluid from the pulmonary intravascular space to the lung interstitial space. Managing volume overload could reduce chronic expansion of the interstitial space in the lungs, which may prevent or slow down the development of lung complications due to CKD. These methods may include lung function testing, biomarkers, and imaging tools such as lung ultrasound.

However, this approach alone will probably not be enough, as understanding the exact mechanisms by which CKD damages the alveolar capillary barrier is essential to developing effective ways to reverse these mechanisms and protect and restore the barrier’s physiological function. These strategies may implicate modulation of the ENaC, Na+/K+/ATPase, AQP1, AQP5, AhR or RAGE among others.

Furthermore, new or more efficient strategies for UT removal are needed. Any gain in efficacy should not come at the cost of an increased harmful impact of the technique itself on the lung; A better HD efficacity in removing UTs should not be counterbalanced by a deeper direct negative impact of this HD technology on the pulmonary structure. The challenge is to find this way of better removing UTs without directly damaging the lung.

Another interesting aspect is to reduce the production of these UTs by making controlled changes to the microbiota or reinforcing the integrity of the intestinal barrier so less endotoxins may pass through to the blood.

In summary, this review outlines the current understanding of the pathophysiological and molecular mechanisms by which UTs and CKD influence the lungs. Overall, the accumulation of UTs in CKD damages endothelial and epithelial cells and the alveolar capillary barrier. The interactions underlying lung-kidney cross-talk in CKD are numerous, and the mechanisms driving these interactions are common across many diseases that affect both organs. Further research is needed to elucidate the mechanisms by which UTs affect the lungs, thereby enabling the development of new therapeutic approaches.

### Limitations

This is a narrative review, and even though we tried our best to include all the studies that examined the impact of UTs on the lungs, there might still be other articles that we did not detect. Furthermore, it was difficult to objectively evaluate the strength of the evidence and compare it across the different studies examined, as they vary in their model and experimental conditions. In addition, much of the current understanding of UTs impact on the lungs is derived from AKI studies, supposing that the physiopathology would probably be the same. This is not necessarily true, and till better direct evidence of UTs effect on the lung would be available, these facts should be considered with caution.

## Figures and Tables

**Table 1:** Main uremic toxins and their characteristics.

Uremic Toxin	Molecular Structure	Molecular Weight	Protein Bound Ratio
Urea	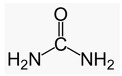	60 Daltons	0%
ADMA	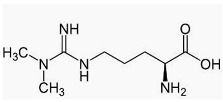	202 Daltons	30%
SDMA	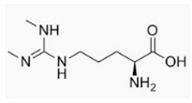	202 Daltons	9%
P-Cresyl Sulfate	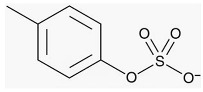	188 Daltons	90–95%
Indoxyl Sulfate	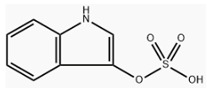	213 Daltons	90%
B2 Microglobulin	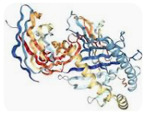	12,000 Daltons	It is a protein

ADMA, Asymmetric Dimethylarginine; SDMA, Symmetric Dimethylarginine.

**Table 2:** A summary of major studies according to the nature of injury and experimental model.

Study	Year	Country	Kidney Injury	Experimental Model	Major Findings
Abbasian et al. [25]	2015	UK	Uremic toxins	Cells	Inorganic phosphate induced cellular stress in endothelial cells.
Ahdieh et al. [26]	2001	USA	Inflammatory factors	Cells	Interleukin IL-4 and IL-13 diminish the capacity of alveolar epithelial cells to maintain barrier function and repair wounds, whereas Interferon IFN-gamma promotes epithelial restitution by enhancing barrier function and wound healing.
Annuk et al. [27]	2001	Estonia	CKD	Humans	Impaired endothelium vasodilation function and oxidative stress are related to each other in patients with CKD.
Bao et al. [28]	2006	USA	Inflammatory factors	Cells	Tumor necrosis factor-alpha, interferon-gamma, and Fas receptor ligation accelerates caspase-3 activation, proteolysis of E-cadherin and beta-catenin, and cellular apoptosis, leading to increased paracellular leak across monolayers of both upper airway and alveolar lung epithelial cultures.
Carter et al. [29]	2021	Germany	CKD	Cells	Uremic toxins promote endothelial maladaptation, VEGF expression and aberrant angiogenesis
Chang et al. [30]	2022	Taiwan	CKD	Humans	CKD patients had higher medial arterial calcification.
Chen et al. [31]	2016	China	AKI	Mice/Cells	The permeability of pulmonary endothelial cells monolayer following exposure to serum from RI/R mice was increased significantly.
De Groot et al. [32]	2004	Germany	CKD	Humans	Renal patients had significantly fewer circulating bone marrow–derived endothelial progenitor cells (EPCs) than healthy subjects.
Dou et al. [33]	2004	France	Uremic toxins	Cells	P-Cresol and Indoxyl Sulfate decrease endothelial proliferation and wound repair.
Dou et al. [34]	2007	Fance	Uremic toxins	Cells	Indoxyl Sulfate enhances ROS production, increases NADPH oxidase activity, and decreases glutathione levels in endothelial cells.
Dou et al. [35]	2015	France	Uremic toxins	Cells	Indole-3 acetic acid (IAA) activated an inflammatory nongenomic aryl hydrocarbon receptor (AhR)/p38MAPK/NF-κB pathway that induced the proinflammatory enzyme cyclooxygenase-2. Additionally, IAA increased production of endothelial reactive oxygen species.
El-Gamal et al. [36]	2012	Austria	Uremic toxins	Cells/Mice	Cyanate compromises endothelial functionality in vitro and in vivo.
Favretto et al. [37]	2017	Brazil	Uremic toxins	Cells	Cell viability decreased after toxin treatment in a dose-dependent manner. Organic anion transporters (OATs) are involved in the uptake of uremic toxins
García-Jérez et al. [38]	2015	Spain	Uremic toxins	Cells	Integrin-linked kinase has a protective effect against endothelial cell damage induced by uremic toxins.
Guo et al. [39]	1998	USA	Nitric Oxide	Cells	Nitric oxide NO at noncytotoxic concentrations decreased Na+ absorption across cultured alveolar epithelial cells monolayers by inhibiting both the amiloride-sensitive Na+ channels and Na(+)-K(+)-ATPase through guanosine 3′,5′-cyclic monophosphate-independent mechanisms.
Gupta et al. [40]	2012	USA	CKD	Humans	Biomarkers of inflammation were inversely associated with measures of kidney function and positively with albuminuria.
Ito et al. [41]	2016	Japan	Uremic toxins	Mice	Aryl hydrocarbon receptor (AhR) mediates Indoxyl Sulfate-enhanced leukocyte–endothelial interactions through activator protein-1 (AP-1) transcriptional activity.
Kim et al. [42]	2017	China	Uremic toxins	Cells	Indoxyl Sulfate mediated immune dysfunction may cause vascular endothelial cell damage.
Komaru et al. [43]	2025	USA	AKI	Mice	Lung capillary neutrophil retention negatively affected oxygenation by causing a ventilation-perfusion mismatch, representing a driver of AKI-induced hypoxemia.
Kramer al. [22]	1999	USA	AKI	Rats	Increased pulmonary vascular permeability develops after isolated renal ischemia/reperfusion injury, and macrophage-derived products are mediators in this response.
Li et al. [44]	2020	China	Uremic toxins	Cells	MicroRNA-214 may protect endothelial cells from damage induced by Indoxyl Sulfate.
Li et al. [45]	2024	China	Uremic toxins	Cells	The expression of tight junction proteins ZO-1 and VE-cadherin was decreased in endothelial cells incubated with uremic toxins.
Ma et al. [46]	2000	USA	Transgenic	Mice	AQP5 is responsible for the majority of water transport across the apical membrane of alveolar epithelial cells. The unimpaired alveolar fluid clearance in AQP5-null mice indicates that high alveolar water permeability is not required for active fluid transport.
Ma et al. [47]	2013	China	AKI	Rat	The TNF-α and IL-6 levels increased significantly and the pulmonary expression of AQP1 and α-ENaC declined at the early stage of AKI.
Martínez-Moreno et al. [48]	2017	Spain	Uremic toxins	Cells	High phosphate (3.3 mmol/L) medium caused an increased expression of the pro-inflammatory mediators intercellular adhesion molecule 1 (ICAM-1), interleukins (ILs) IL-1β, IL-6, IL-8 and tumor necrosis factor α (TNF-α), as well as an increase in reactive oxygen/nitrogen species (ROS/RNS) production. This was accompanied by the activation of nuclear factor κ-light-chain-enhancer of activated B cells (NF-κB) signaling.
Masai et al. [49]	2010	Japan	Uremic toxins	Cells	Indoxyl Sulfate enhanced reactive oxygen species (ROS) production, induced the expression of MCP-1, and activated NF-κB. IS increases NADPH oxidase-derived ROS, which in turn, activates the MAPK/NF-κB pathway and leads to induction of MCP-1
Merino et al. [50]	2010	Spain	CKD	Humans	Microinflammatory status is promoting the endothelial damage in dialysis patients.
Mukai et al. [7]	2018	Sweden	CKD	Humans	Lung dysfunction is a common complication in patients with advanced CKD
Nemmar et al. [51]	2017	UAE	CKD	Mice	CKD is accompanied by lung oxidative stress, DNA damage, apoptosis, and Nrf2 expression and fibrosis.
Nowak et al. [52]	2020	USA	CKD	Humans	Vascular oxidative stress is present in CKD.
Peng et al. [53]	2011	USA	Uremic toxins	Cells	Both hyperphosphatemia and hypophosphatemia decrease eNOS expression and NO production and are associated with endothelial cell death.
Rabb et al. [23]	2003	USA	AKI	Rats	Ischemic acute renal failure leads to down regulation of pulmonary ENaC, Na, K-ATPase and aquaporin-5, but not aquaporin-1. Since bilateral nephrectomy but not single kidney I/R injury also leads to lung changes, these changes are likely mediated by uremic toxins, rather than reperfusion products.
Saum et al. [54]	2018	USA	Uremic serum	Cells	Krüppel-like factor 2 (KLF2), a key regulator of endothelial function and activation, is suppressed in uremic milieu, which may exacerbate endothelial dysfunction.
Shuto et al. [55]	2009	Japan	Uremic toxins	Cells/Rats/ Humans	Phosphorus load increased production of endothelial cells reactive oxygen species and decreased nitric oxide production. Phosphorus loading inhibited endothelium-dependent vasodilation of rat aortic rings. The high dietary phosphorus load significantly decreased flow-mediated dilation.
Six et al. [56]	2014	France	Uremic toxins	Cells	Klotho deficiency is deleterious to vascular smooth muscle and endothelium whereas Klotho sufficiency is protective against the negative effects of phosphate and FGF23.
Vaziri et al. [57]	2013	USA	Uremic toxins	Cells	Exposure to uremic milieu damages the intestinal epithelial TJ and impairs its barrier function
Verkman et al. [58]	2000	USA	Transgenic	Mice	Deletion of AQP1 or AQP5, water channels in lung endothelia and epithelia, resulted in a 90% decrease in airspace-capillary water permeability.
Ware et al. [59]	2001	USA	ALI	Humans	Alveolar fluid clearance in patients with ALI is impaired
Watanabe et al. [60]	2015	Japan	Uremic toxins	Cells	p-Cresyl Sulfate enhances the production of reactive oxygen species ROS in vascular endothelial and smooth muscle cells.
Wu et al. [61]	2008	China	Uremic serum	Cells	Serum of uremia patients increased the permeability of pulmonary endothelial cells.
Yabuuchi et al. [62]	2016	Japan	AKI	Rat	AKI causes dysregulation of pulmonary AQP-5 expression, in which Indoxyl Sulfate could play a toxico-physiological role.
Yang et al. [63]	2012	China	Uremic toxins	Cells	The release of reactive oxygen species ROS and the expression of monocyte chemoattractant protein-1 (MCP-1) were enhanced while the cell viability and production of nitric oxide (NO) were inhibited by Indoxyl Sulphate, while the phosphorylation of p38MAPK and the nuclear translocation of NF-κB were increased. Klotho protein has the ability to ameliorate the IS-induced endothelial dysfunction, which may be partly through inhibiting the ROS/p38MAPK and downstream NF-κB signaling pathways.
Yuan et al. [64]	2013	Sweden	CKD	Humans	Vascular endothelial growth factor (VEGF) and its soluble receptor 1 (sVEGFR-1) are associated with biomarkers of inflammation.
Zarbock et al. [65]	2006	Germany	AKI	Mice	Pulmonary recruitment of uremic neutrophils was significantly attenuated compared with that of normal neutrophils in aseptic ALI.
Zhao et al. [66]	2015	UK	AKI	Rats	Renal graft injury triggered remote lung injury, likely through regulated necrosis.
Zickler et al. [67]	2018	Germany	CKD	Cells	Uremic serum contains higher levels of uremic toxins TNF-α and IL-6 that promotes vascular calcification through a signaling pathway involving TNF-α, IL-6 and the AP-1/c-FOS cytokine-signaling axis.

ALI: Acute lung injury; AKI: Acute kidney injury; AQP1: Aquaporin 1; AQP5: Aquaporin 5; CKD: Chronic kidney disease; ENaC: Epithelial sodium channel; IL: Interleukin; MAPK: Mitogen-activated protein kinases; NADPH oxidase: Nicotinamide adenine dinucleotide phosphate oxidase; NF-κB: Nuclear factor kappa-light-chain-enhancer of activated B cells; Nrf2: Nuclear erythroid-related factor 2; TJ: Tigh junction; TNF: Tumor necrosis factor; VEGF: Vascular endothelial growth factor.

**Table 3:** Molecular changes in the alveoli in uremic conditions and their associated markers.

Molecular Structure	Location	Function	Changes Due to UTs	Experimental Conditions	Experimental Model	Study
ENaC	Apical membrane of the alveolar epithelial cell	Participating in creating a sodium concentration gradient	Reduced	AKI	Rats	Rabb et al. [23]
			Reduced	AKI	Rats	Ma et al. [47]
Na+/K+/ATPase	Basolateral membrane of the alveolar epithelial cell	Participating in creating a sodium concentration gradient	Reduced	AKI	Rats	Rabb et al. [23]
AQP5	Apical membrane of the alveolar epithelial cell	Facilitate water movement through the alveolar epithelial cell	Reduced	AKI	Rats	Rabb et al. [23]
			Reduced	AKI	Rats	Yabuuchi et al. [62]
AQP1	Membrane of the pulmonary endothelial cell	Facilitate water movement through the pulmonary endothelial cell	Unchanged	AKI	Rats	Rabb et al. [23]
			Reduced	AKI	Rats	Ma et al. [47]
TJ	Linking between cells	Regulate the iter-cellular fluid movements	Reduced	Uremic toxins	Cells	Vaziri et al. [57]

AQP1: Aquaporin 1; AQP5: Aquaporin 5; ENaC: Epithelial sodium channel; Na+/K+/ATPase: Sodium-potassium ATPase channel.

## Data Availability

No new data were created or analyzed in this study.

## Supplementary Materials

The following supporting information can be downloaded at: https://www.mdpi.com/article/10.3390/toxins18030126/s1, Table S1: Search terms and number of results in PubMed which were used to elaborate the review.

## Abbreviations

ADMA	Asymmetric Dimethylarginine
AGEs	Advanced glycation end products
AhR	Aryl hydrocarbon receptor
ALI	Acute lung injury
AKI	Acute kidney injury
AQPs	Aquaporins
CKD	Chronic kidney disease
CRP	C-reactive protein
EMPs	Endothelial microparticles
ENaC	Epithelial sodium channel
eNOS	Endothelial nitric oxide synthase
EPC	Endothelial progenitor cell
ESKD	End-stage kidney disease
FGF-23	Fibroblast growth factor
GSTM-1	Glutathione S-transferase-1
HD	Hemodialysis
IAA	Indole-3-Acetic acid
ICAM-1	Intercellular adhesion molecule-1
IFN-γ	Interferon gamma
IL-1	Interleukin-1
IL-6	Interleukin-6
IS	Indoxyl sulfate
JAMs	Junctional adhesion molecules
MAPK	Mitogen-activated protein kinases
MCP-1	Monocyte chemoattractant protein-1
MIP-1	Macrophage inflammatory protein
NADPH oxidase	Nicotinamide adenine dinucleotide phosphate oxidase
Na+/K+/ATPase	Sodium-potassium ATPase
NF-κB	Nuclear factor kappa-light-chain-enhancer of activated B cells
NO	Nitric oxide
Nrf2	Nuclear erythroid-related factor 2
OATs	Organic anion transporters
PCS	p-Cresyl Sulfate
PD	Peritoneal dialysis
PECAM-1	Platelet endothelial cell adhesion molecule-1
PMVEC	Pulmonary microvascular endothelial cell
RAGE	Receptor for advanced glycation endproducts
RANK	Receptor activator of nuclear factor
RANKL	Receptor activator of nuclear factor ligand
RIR	Renal ischemia–reperfusion
ROS	Reactive oxygen species
RNS	Reactive nitrogen species
SDMA	Symmetric dimethylarginine
TGF-β	Transforming Growth Factor Beta
TJ	Tight Junction
TMAO	Trimethylamine N-oxide
TNF	Tumor necrosis factor
TNF-α	Tumor necrosis factor-alpha
ULI	Uremic lung injury
UT	Uremic toxins
VE-cadherin	Vascular endothelial cadherin
VEGF	Vascular endothelial growth factor
ZO-1	Zonula occludens-1
