# Supplementary material for: Uremic Toxins and the Lung Alveolar Capillary Barrier: A Narrative Review
Source: Toxins (Basel). 2026 Mar 2;18(3):126. doi: 10.3390/toxins18030126 (PMC13030341; doi:10.3390/toxins18030126)
Supplement: Supplementary file 1 [file toxins-18-00126-s001.zip › toxins-4126733-supplementary.pdf]

Supplementary Materials : Uremic Toxins and the Lung Alveolar Capillary Barrier: A Narrative Review

Saleh Kaysi, Maxime Taghavi, Alissa El Mourabi, Marie-Hélène Antoine, Eric De Prez and Joëlle Nortier

Table S1 : Search terms and number of results in PubMed which were used to elaborate the review

Term	Number of results
((uremia[Title]) AND (oxidative[Title])) OR (((((((uremia[Title]) AND (endothelial[Title])) OR ((uremia[Title]) AND (epithelial[Title]))) OR ((uremia[Title]) AND (pulmonary[Title]))) OR ((uremia[Title]) AND (lung[Title]))) OR ((uremic toxins[Title]) AND (epithelial[Title]))) OR ((uremic toxins[Title]) AND (endothelial[Title])))	122
((((renal failure[Title]) AND (inflammation[Title])) OR ((end stage kidney disease[Title]) AND (inflammation[Title]))) OR ((uremia[Title]) AND (inflammation[Title]))) OR ((uremic toxins[Title]) AND (inflammation[Title]))	139
(((((((((barrier[Title]) AND (renal[Title])) OR ((alveolar[Title]) AND (renal[Title]))) OR ((barrier[Title]) AND (uremic[Title]))) OR ((barrier[Title]) AND (kidney[Title]))) OR ((pulmonary[Title]) AND (uremic[Title]))) OR ((pulmonary[Title]) AND (kidney[Title]))) OR ((lung cells[Title]) AND (kidney[Title]))) OR ((lung[Title]) AND (uremic[Title]))) OR ((alveolar[Title]) AND (uremic[Title]))) OR ((alveolar[Title]) AND (kidney[Title]))	1028
